# Statistical shape modelling to aid surgical planning: associations between surgical parameters and head shapes following spring-assisted cranioplasty

**DOI:** 10.1007/s11548-017-1614-5

**Published:** 2017-05-26

**Authors:** Naiara Rodriguez-Florez, Jan L. Bruse, Alessandro Borghi, Herman Vercruysse, Juling Ong, Greg James, Xavier Pennec, David J. Dunaway, N. U. Owase Jeelani, Silvia Schievano

**Affiliations:** 1 UCL Great Ormond Street Institute of Child Health, 30 Guilford Street, London, WC1N 1EH UK; 2 Craniofacial Unit, Great Ormond Street Hospital for Children NHS Foundation Trust, London, UK; 3 Centre for Cardiovascular Imaging, UCL Institute of Cardiovascular Science, London, UK; 4 Asclepios Team, Inria, Sophia Antipolis, France

**Keywords:** Craniofacial surgery, Partial least squares regression, Statistical shape modelling, 3D scanning, Craniosynostosis, Clinical decision Support

## Abstract

**Purpose:**

Spring-assisted cranioplasty is performed to correct the long and narrow head shape of children with sagittal synostosis. Such corrective surgery involves osteotomies and the placement of spring-like distractors, which gradually expand to widen the skull until removal about 4 months later. Due to its dynamic nature, associations between surgical parameters and post-operative 3D head shape features are difficult to comprehend. The current study aimed at applying population-based statistical shape modelling to gain insight into how the choice of surgical parameters such as craniotomy size and spring positioning affects post-surgical head shape.

**Methods:**

Twenty consecutive patients with sagittal synostosis who underwent spring-assisted cranioplasty at Great Ormond Street Hospital for Children (London, UK) were prospectively recruited. Using a nonparametric statistical modelling technique based on mathematical currents, a 3D head shape template was computed from surface head scans of sagittal patients after spring removal. Partial least squares (PLS) regression was employed to quantify and visualise trends of localised head shape changes associated with the surgical parameters recorded during spring insertion: anterior–posterior and lateral craniotomy dimensions, anterior spring position and distance between anterior and posterior springs.

**Results:**

Bivariate correlations between surgical parameters and corresponding PLS shape vectors demonstrated that anterior–posterior (Pearson’s $r=0.64, p=0.002$) and lateral craniotomy dimensions (Spearman’s $\rho =0.67, p<0.001$), as well as the position of the anterior spring ($r=0.70, p<0.001$) and the distance between both springs ($r=0.67, p=0.002$) on average had significant effects on head shapes at the time of spring removal. Such effects were visualised on 3D models.

**Conclusions:**

Population-based analysis of 3D post-operative medical images via computational statistical modelling tools allowed for detection of novel associations between surgical parameters and head shape features achieved following spring-assisted cranioplasty. The techniques described here could be extended to other cranio-maxillofacial procedures in order to assess post-operative outcomes and ultimately facilitate surgical decision making.

## Introduction

Craniosynostosis is a congenital condition characterised by premature fusion of one or more cranial sutures during infancy. This can result in aesthetic and/or functional problems due to skull growth restrictions, often requiring early surgical intervention to reshape the skull [1–3]. In this context, assessment of head shape features is essential to drive craniosynostosis management and inform treatment choice. However, often this analysis relies only on clinician experience and expertise, with the addition of a few linear measurements and no objective way to assess the overall three-dimensional (3D) head shape characteristics and abnormalities [3–8].

**Fig. 1:**
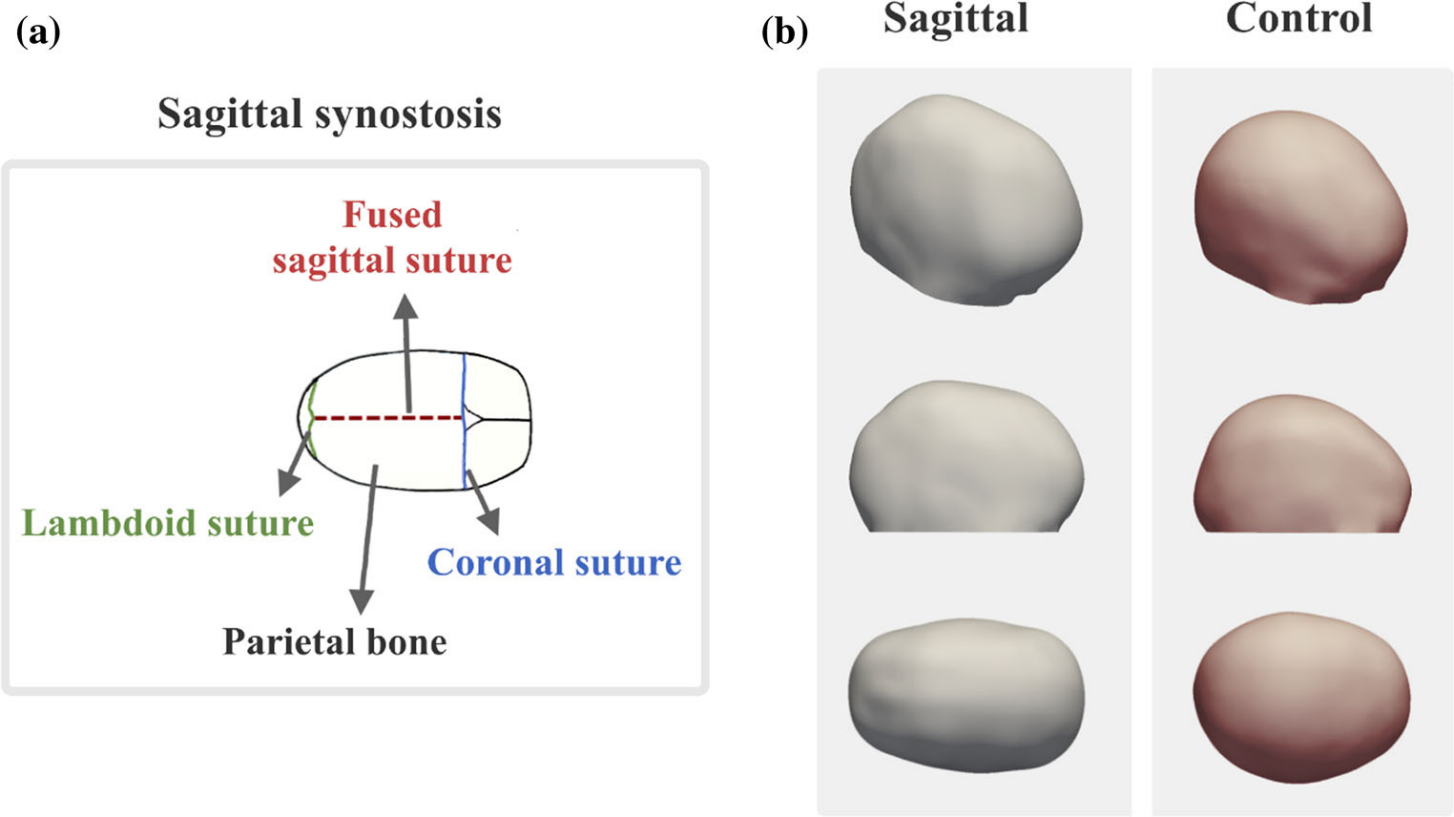
Pathology and head shape features associated with sagittal synostosis. **a** Schematic of an infant skull with sagittal synostosis viewed from above. The coronal and lambdoid sutures are patent while the sagittal suture is fused. **b** 3D surface head scans of a sagittal patient and an age-matched control, showing the 3D, lateral and top view for each case. The sagittal patient has a narrower and longer head shape, wider anteriorly than posteriorly, when compared to the control shape

In recent years, computer-assisted analysis of 3D medical image data has been employed to improve the head shape description [9–12] and to aid in the diagnosis of craniosynostosis [13–19] as well as in the evaluation of surgical outcomes [20–23]. With the aim of facilitating cranio-maxillofacial surgery, statistical shape modelling (SSM) has been used in virtual surgery planning [24–28] as well as in the design of pre-fabricated templates [29–31], surgical guides [32, 33] and cranial implants [34, 35] to achieve desired patient-specific post-operative outcomes on-table. However, some craniofacial procedures rely on gradual post-operative skull remodelling instead of acute changes and thus seek to obtain desired shapes not immediately on the operative table, but months later.

**Fig. 2:**
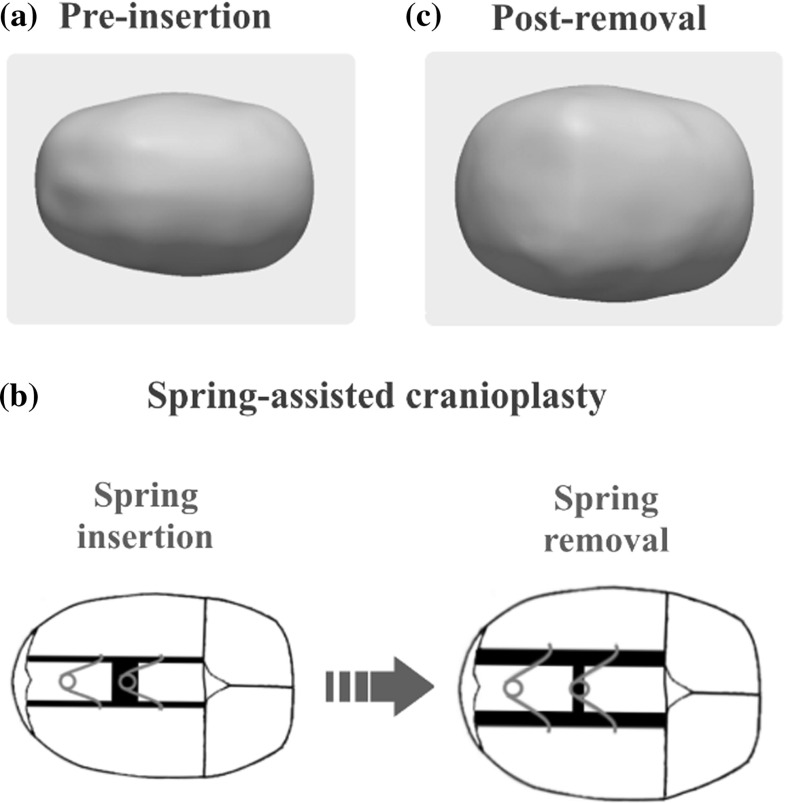
Outline of head shape changes induced by spring-assisted cranioplasty on a patient with sagittal synostosis. **a** Top view showing a long and narrow head shape before insertion. **b** Schematic of spring-assisted cranioplasty: two metal springs are placed in the parietal bone, which open gradually pushing the skull to widen. **c** Top view of the head scan after spring removal indicating a bigger ratio between head width and length when compared to the pre-insertion head shape

**Fig. 3:**
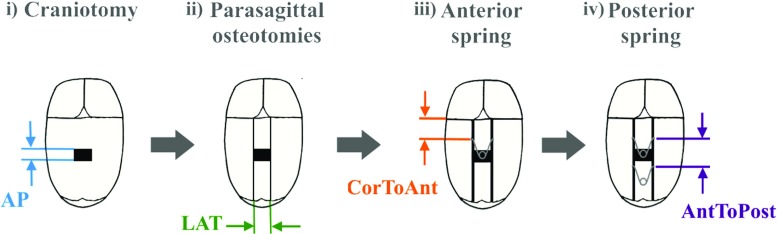
Representation of spring insertion surgery indicating the recorded parameters. **i** A rectangular craniotomy is first performed and **ii** two parasagittal osteotomies are made. The **iii** anterior and **iv** posterior springs are then placed on each side of the osteotomy. The recorded parameters include the anterior–posterior (*AP*) and lateral (*LAT*) dimensions of the craniotomy, the distance from the coronal suture to the anterior spring (*CorToAnt*) and the distance between anterior and posterior springs (*AntToPost*)

One of these procedures is spring-assisted cranioplasty (SAC), used to correct the head shape in infants with sagittal synostosis [36–38], where the sagittal suture on top of the head fuses prematurely (Fig. 1a). Sagittal synostosis is the most common presentation of single suture craniosynostosis [5, 39] and results in abnormal skull growth leading to long and narrow heads, often with a bullet-like shape with the posterior part of the head being narrower than the anterior, best appreciated when viewed from above (Fig. 1b) [5, 39–41]. Corrective surgery via SAC [37] involves osteotomies and the temporary placement of spring-like metallic distractors, which are left on the patient to gradually expand, driving the skull to widen over subsequent weeks and months (Fig. 2). Approximately 4–5 months after insertion, the springs are removed with a second short procedure. Surgical parameters, such as osteotomy and spring positions, are among the factors expected to influence overall and localised head remodelling. However, due to the dynamic nature of the procedure, predicting the effects of on-table surgical choices on future head shape outcomes is not always straightforward [36], thus sometimes resulting in suboptimal remodelling. To the best of the authors’ knowledge, no studies to date have applied advanced 3D computational tools to analyse associations between SAC parameters that can be altered during surgery and long-term head shape outcomes.

In this study, a nonparametric SSM technique [42–44] was employed to unveil population-based associations between those variables depending on the surgeon choice at the time of spring insertion, and global and regional 3D head shape features months later when springs are removed. The surgical parameters that the surgeon can control when operating were recorded during spring insertion and head shapes of sagittal patients after spring removal were captured using non-invasive 3D handheld surface scanning [20]. Partial least squares regression (PLS) [45] was then employed to extract 3D head shape features most associated with each of the recorded surgical parameters [42–46].

Population-based analysis of 3D post-operative medical image data using computational SSM tools was expected to detect and untangle novel associations between each of the surgical parameters and the achieved head shape outcomes, which may ultimately impact on surgical decision making.

## Materials and methods

### Patient population

Twenty consecutive patients with non-syndromic, single suture, sagittal synostosis (17 male) who underwent SAC at Great Ormond Street Hospital for Children (GOSH, London, UK) were prospectively recruited for this study between May 2015 and 2016. Patient age at time of spring insertion was 5.2 ± 1.2 months and springs were removed when the patients were 9.5 ± 1.4 month old. Written parental consent was obtained during pre-operative clinic for all patients for acquisition of 3D head scans and their use in research.

### Surgical technique and recorded parameters

Clinical details about SAC (Figs. 2, 3) can be found in Rodgers et al. [37]. A schematic of spring insertion surgery is shown in Fig. 3. Spring insertion is performed with the patient in the prone position around mid-way between the coronal and lambdoid sutures through one small transverse scalp incision. Once the bone is exposed, a rectangular craniotomy is made and the small piece of parietal bone is discarded. Starting from the craniotomy, two osteotomies are made parallel to the fused sagittal suture extending from the coronal to lambdoid sutures, leaving the bone with the fused sagittal suture in place. Two standardised metal springs (Active Spring Company, Thaxted, UK) are then inserted into notches made in the parietal bone on each side of the osteotomy to push the edges apart and remodel the head shape.

Figure 3 shows the parameters that were recorded during spring insertion in order to quantify the surgical steps described above: the size of the rectangular craniotomy defined by the anterior–posterior (*AP*) and the lateral (*LAT*) lengths; the distance from the coronal suture to the anterior spring (*CorToAnt*) as well as the distance between the anterior and posterior springs (*AntToPost*).

### 3D head scans

Since sagittal patients do not routinely undergo computed tomography scanning at our centre [47, 48], 3D head scans were acquired in theatre immediately before spring insertion (*pre-insertion*, Fig. 2a) and immediately after removal (*post-removal*, Fig. 2c) using a 3D handheld surface scanner (M4D Scanner, Rodin4D, Pessac, France). Detailed description of scan acquisition and post-processing can be found in Tenhagen et al. [20]. Briefly, scans were exported as 3D computational surface meshes in stereolithography (STL) format, and post-processed to clean artefacts and isolate the region of interest (i.e. calvarium) by manually cutting a plane through the nasion and both tragion points in MeshMixer (Autodesk Inc., Toronto, Canada). Post-removal 3D scans were rigidly registered with the *N*-point registration algorithm in 3-matic (Materialise, Leuven, Belgium) using the same landmarks as for the cutting plane. The registered scans were then used for statistical shape modelling.

Linear measurements were automatically computed on the STL files using the “meshcube” function in the Morpho-package of R (v.3.3.0, R Foundation for Statistical Computing, Vienna, Austria). This function calculates the corners of the bounding box comprising the STL mesh, which can then be translated to head width, length and height measurements.

Pre-operative head width and length were used to normalise the recorded surgical parameters according to head size. *AP*, *CorToAnt* and *AntToPost* were normalised as percentages of pre-insertion head length, while *LAT* was represented as a percentage of pre-insertion head width, as indicated in Eqs. 1–4:

1 $$\begin{aligned}&{} \textit{AP}\left( \% \right) = \frac{\textit{AP}}{\textit{Head length}} \end{aligned}$$

2 $$\begin{aligned}&{} \textit{LAT}\left( \% \right) = \frac{\textit{LAT}}{\textit{Head width}} \end{aligned}$$

3 $$\begin{aligned}&{} \textit{CorToAnt}\left( \% \right) = \frac{\textit{CorToAnt}}{\textit{Head length}} \end{aligned}$$

4 $$\begin{aligned}&{} \textit{AntToPost}\left( \% \right) = \frac{\textit{AntToPost}}{\textit{Head length}} \end{aligned}$$

### Statistical shape modelling and partial least squares regression

Statistical shape analysis was performed to assess how surgical parameters at spring insertion (Fig. 3) related to head shape variability when springs were removed.

**Table 1:** Average values and standard deviations (SD) of head morphometric parameters measured on post-removal 3D head scans of the population and on the computed post-removal template

	Volume ($\hbox {cm}^{3}$)	Width (mm)	Length (mm)	Height (mm)
$\hbox {Average of the population} \pm \hbox {SD}$	1536 ± 104	127 ± 3	174 ± 6	117 ± 4
Computed post-removal template	1546	128	175	117
Deviation ($\%$)	−0.65	−0.79	−0.57	<0.01

All deviations between the population average and the computed template shape are within $\pm 1$%

Based on the twenty previously registered post-removal 3D head scan computational surface meshes, a post-removal template shape $T_\mathrm{post}$ (i.e. anatomical 3D mean head shape after spring removal) was computed. Specifically, the nonparametric statistical shape modelling framework Deformetrica (www.deformetrica.org) [42–44] was used to simultaneously compute ${T}_\mathrm{post}$ and the associated patient-specific deformation functions $\varPhi _{i}$ by registering ${T}_\mathrm{post}$ to each subject shape *i* [49]. Within Deformetrica, shapes are modelled as *mathematical currents* [50], which are surrogate representations of shapes that enable analysis without landmarking, thus making this method attractive for smooth, landmark-poor shapes such as the calvarium [20, 51]. For the current-based analysis, input shapes and the deformation functions $\varPhi _{i}$ need to be defined in vector spaces, generated by Gaussian kernels as detailed in [42–44]. Gaussian kernel widths $\lambda _{W,}$ for shape, and $\lambda _{V,}$ for deformation parameterisation, were here set to 10 and 30 mm, respectively, following protocols described in Bruse et al. [42]. Each patient head shape was then expressed as a deformation towards the template shape $\varPhi _{i}\;(T_\mathrm{post})$ and numerically parameterised by a set of deformation vectors $\beta _{i}$. All $\beta _{i}$ together constitute a deformation matrix *M*, which parameterises all 3D head shape feature information based on the common basis shape ${T}_\mathrm{post}$. *M* allows extraction, quantification and visualisation of dominant 3D shape features most associated with a chosen response parameter via partial least squares regression (PLS) [42, 51].

PLS was used here to extract *PLS shape modes *[45], which represent the dominant post-removal head shape features *most correlated* with the surgical parameters of interest [42, 43, 52]. First, in order to focus predominantly on 3D head shape features and not on head size, shape features most related to post-removal head volume $V_{post }$ were extracted and size effects caused by differences in volume among the patients were removed by using the residuals of this calculation as basis for all further PLS runs, as detailed in [42, 52]. Afterwards, PLS shape modes most related to recorded surgical parameters (normalised *AP*, *LAT*, *CorToAnt*, *AntToPost*) were extracted. Further, each patient head shape was projected onto the respective PLS shape mode in order to obtain a *PLS shape vector *(scalar product between $\varPhi _{i }$ and shape mode), which is a low-dimensional numerical representation of how much of the respective shape mode 3D features are contained within each patient head shape [42, 51]. The *PLS shape vectors* were then used for further bivariate correlation analyses. Head shape features most related with a chosen response parameter were visualised in Paraview [53] as deformations of ${T}_\mathrm{post}$ along the respective PLS shape mode, towards large ($+$3 SD) and small (−3 SD) values of the response parameter. Such 3D models of the extreme cases were used to describe the most relevant shape features concerning the correction of long, narrow and bullet-like head shapes characteristics of sagittal synostosis.

The computed template was validated via leave-one-out cross-validation and geometric morphometry, as described in Bruse et al. [42]. Head volume, width, length and height were measured on all post-removal 3D scans ($n=20$) as well as on ${T}_\mathrm{post}$, with the volume confined within the mesh surface and the horizontal plane considered as the head volume and calculated using the vascular modelling toolkit (VMTK, Orobix, Bergamo, Italy) [54] in combination with MATLAB (The MathWorks, Inc., Natick, MA, USA). Percentage differences between average measurements of the population and measurements taken from the post-removal template were computed to assess whether ${T}_\mathrm{post}$ was an acceptable mean shape representation of the population.

### Statistical analysis

Mean values and standard deviations ($\hbox {mean}\pm \hbox {SD}$) were calculated for head volume, width, length and height measured on the 3D scans, as well as for the recorded surgical parameters. Associations between *PLS shape vectors* most related to differences in normalised *AP*, *LAT*, *CorToAnt *and *AntToPost* (after removing size effects) and the corresponding surgical parameter were evaluated via bivariate correlation analyses. Normality of the data was assessed using Shapiro–Wilk test. For parametric data, Pearson’s *r* correlation coefficient was used, while Spearman’s $\rho$ was employed for nonparametric data. In order to detect influential observations in the PLS regression, Cook’s distance ${D}_\mathrm{Cook}$ [55] was calculated for each PLS regression run and when ${D}_\mathrm{Cook}$ exceeded four times the mean, these data were excluded from the analysis. Correlations were considered statistically significant for *p* values <0.05. All statistical analyses were performed using R.

## Results

### Post-removal template

Based on the 3D surface head scans of the recruited sagittal patients after the removal of springs, the post-removal head template was computed. Comparisons of head volume, width, length, and height measurements performed on the population and on the template show that all deviations were within $\pm 1\%$ (Table 1); hence, ${T}_\mathrm{post}$ was considered a good representation of the population average.

### Normalised surgical parameters

Population values of normalised surgical parameters recorded during spring insertion are reported in Table 2. On average (±SD), the craniotomy had a rectangular size of 9 ± 2% (*AP*) by $18\pm 3\%$ (*LAT*). The first spring was located at a distance of 31 ± 4% from the coronal suture, while the average distance between the anterior and posterior springs was $22\pm 6\%$, both shown as percentages of head length. Among all parameters, the distance between the springs showed most variability, as it ranged from 14 to $35\%$ of head length.

**Table 2:** Average, standard deviations (±SD) and minimum and maximum (min–max) values of normalised surgical parameters recorded during spring insertion (Fig. 3)

	Average ± SD (min–max)
*AP* ($\%$)	9 ± 2 (6–12)
*LAT* ($\%$)	18 ± 3 (11–21)
*CorToAnt* ($\%$)	31 ± 4 (22–38)
*AntToPost* ($\%$)	22 ± 6 (14–35)

Anterior–posterior craniotomy size (*AP*) as well as distances from the coronal suture to the anterior spring (*CorToAnt*) and anterior to posterior springs (*AntoToPost*) are shown as percentages of pre-insertion head length, while the lateral craniotomy size (*LAT*) is shown as a percentage of pre-insertion head width

**Fig. 4:**
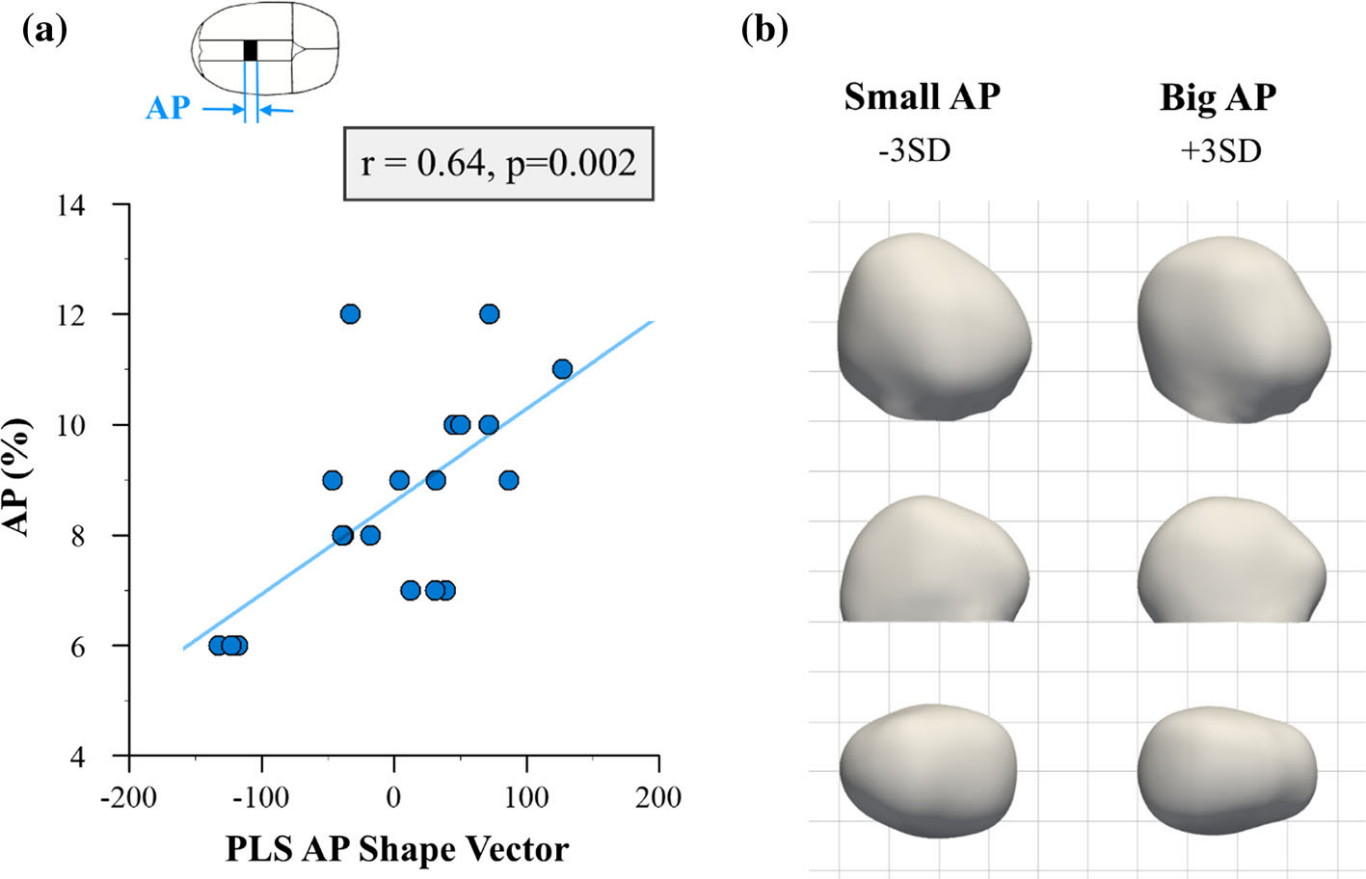
Partial least squares analysis of anterior–posterior craniotomy size (*AP*). **a** Correlation between *PLS AP shape vector* and surgical parameter *AP* showing a strong association. **b** 3D, lateral and top views of computed template shape deformed along the *PLS AP shape mode* for small and big values of *AP* (±3 SD), showing that big values of *AP* are associated with bigger bi-parietal widening

**Fig. 5:**
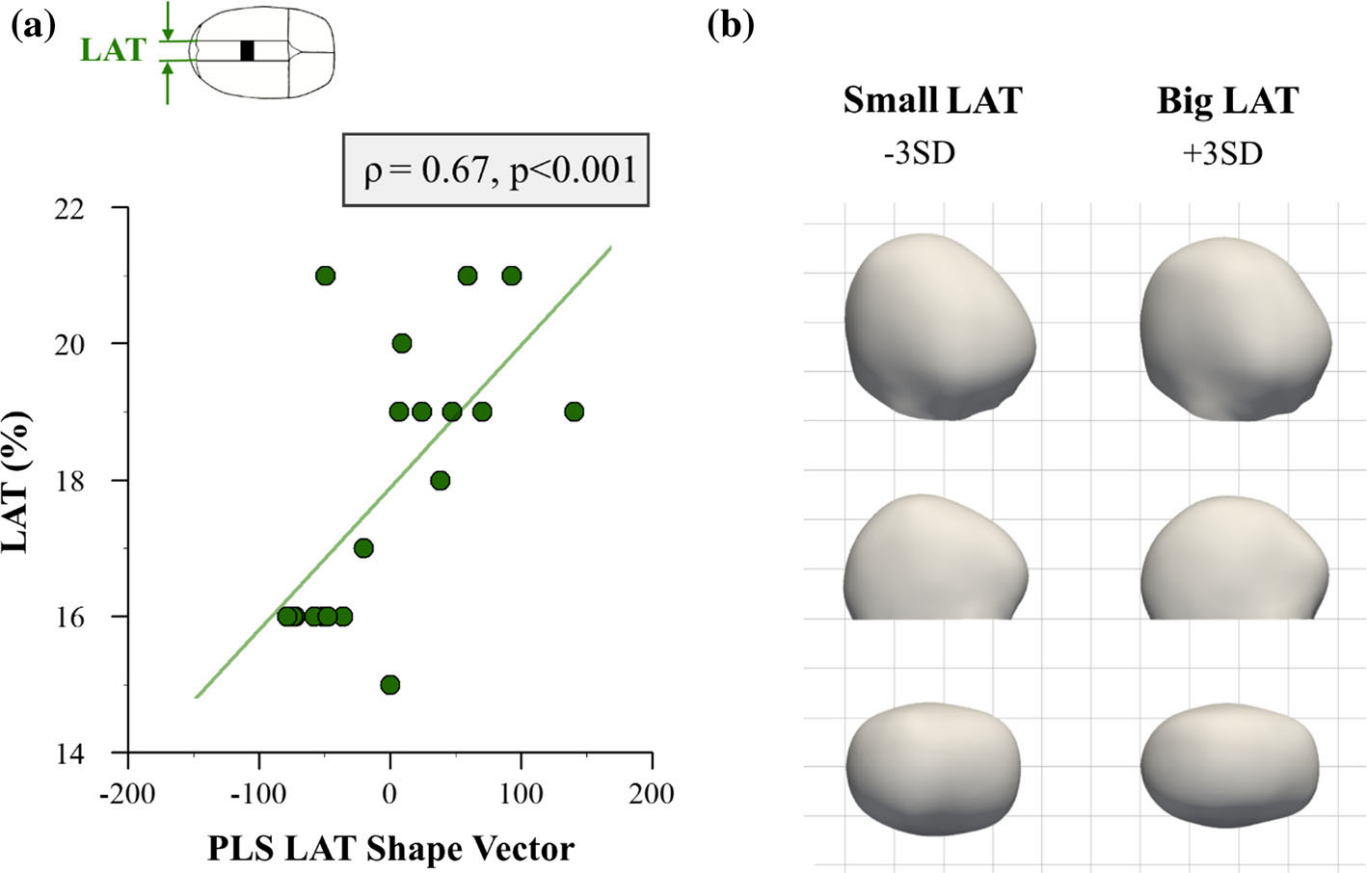
Partial least squares analysis of lateral osteotomy width (*LAT*). **a** Correlation between *PLS LAT shape vector* and *LAT* parameter. **b** 3D, lateral and top views of computed statistical shape models for small and big values of *LAT* (±3 SD), indicating that big values of*LAT* are associated with longer and narrower head shapes

**Fig. 6:**
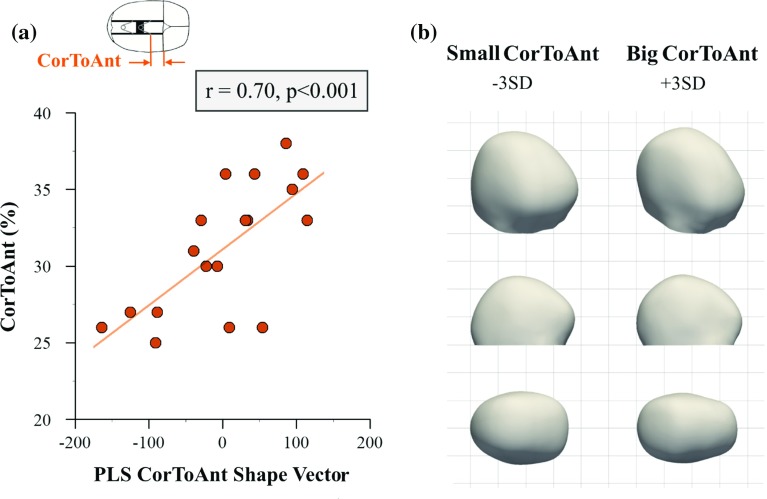
Partial least squares analysis of the distance between coronal suture and anterior spring (*CorToAnt*). **a** Correlation between *PLS CorToAnt shape vector* and *CorToAnt* parameter. **b** 3D, lateral and top views of computed statistical shape models for small and big values of *CorToAnt* (±3 SD), illustrating that positioning the anterior spring further from the coronal suture leads to bigger bi-parietal widening

**Fig. 7:**
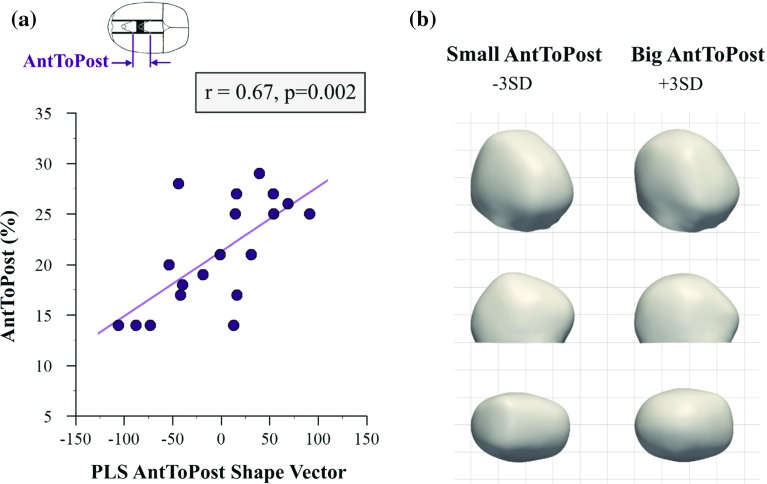
Partial least squares analysis of the distance between anterior and posterior springs (*AntToPost*). **a** Correlation between *PLS AntToPost shape vector* and *AntToPost* surgical parameter. **b** 3D, lateral and top views of computed statistical shape models for small and big values of *AntToPost* (±3 SD), revealing that positioning springs close to each other leads to localised head shape changes

### PLS results

Initial PLS analysis, regressing 3D head shape with post-removal head volume $V_\mathrm{post}$, accounted for $17\%$ of 3D shape response ($n=20$, Pearson’s $r=0.95, p<0.001$) and was used to remove size effects from the subsequent regression analyses.

Figures 4–7 show correlations between the analysed parameters (normalised *AP*, *LAT*, *CorToAnt *and *AntToPost*) and their corresponding *PLS shape vectors* after accounting for volume differences. In addition, computed 3D models for the extreme cases of small (−3 SD) and big ($+$3 SD) values of the corresponding surgical parameter are displayed as deformations of the computed template head shape along the respective PLS shape modes for each of the surgical parameters. One subject had to be removed following the Cook’s distance analysis, for each of the regressions.

*AP* accounted for $11\%$ of the shape response. As shown in Fig. 4a, *AP* and its *PLS shape vector* were significantly correlated ($n=19$, Pearson’s $r=0.64, p=0.002$). Small values of *AP* were associated with bullet-like post-removal head shapes (wider anteriorly than posteriorly) with a prominence on top of the head, whereas big *AP* values were related to bigger bi-parietal widening (Fig. 4b), focused more centrally and posteriorly.

The *PLS shape vector* of the lateral dimension of osteotomies was correlated with the *LAT* parameter ($n=19$, Spearman’s $\rho =0.67, p<0.001$) and accounted for $8.1\%$ of the shape response (Fig. 5a). Bigger values of *LAT* were associated with longer and narrower head shapes (Fig. 5b).

*CorToAnt*, which accounted for $11\%$ of shape response, was strongly correlated with the corresponding shape vector ($n=19, r=0.70, p<0.001$) (Fig. 6a). Positioning the anterior spring further from the coronal suture (big values of *CorToAnt*) was associated with bigger bi-parietal widening of the posterior part of the head (Fig. 6b) with also a more rounded posterior profile, opposite to the shape obtained when positioning the spring more anteriorly.

*AntToPost* was significantly correlated with the corresponding shape vector ($n=19, r=0.67, p=0.002$) and accounted for $7.3\%$ of shape response (Fig. 7a). Having both springs close to each other (small values of *AntToPost*) was associated with localised prominences on top of the head, while positioning both springs further apart led to more globalised widening of the head with a more rounded profile in the back of the head (Fig. 7b).

## Discussion

Spring-assisted cranioplasty is employed to correct the head shape of children with sagittal synostosis, who have long, narrow and sometimes bullet-like skulls wider anteriorly than posteriorly. SAC is a minimally invasive technique which relies on the gradual opening of spring-like distractors to push the skull to widen over time [36–38, 56]. Due to the complex dynamic biomechanical remodelling, the effects of surgical choices (i.e. craniotomy size and spring positioning) on head widening several months after the operation are difficult to foresee [36]. To date, the reasons behind differences in achieved head shape outcomes, with occasional suboptimal results, remain unclear [37].

In the current study, population-based statistical shape modelling was used to understand how each of the surgical parameters (currently used at GOSH for SAC) affects global and local post-surgical head shape outcome. Combining non-invasive 3D head shape scanning with nonparametric statistical shape modelling and PLS regression, it was found that craniotomy dimensions and positions of springs on average had significant effects on head shapes achieved at the time of spring removal. While previous studies have used SSM to predict the shape of missing anatomical bony parts [27, 34] or choose best suited bone segments to plan patient-specific mandibular reconstructions [28], to the best of our knowledge, this is the first prospective study establishing direct population-based associations between craniofacial surgical choices and long-term head shape outcomes.

Specifically, we computed a 3D template head shape based on a cohort of SAC patients and employed PLS to quantify and visualise trends of localised head shape changes associated with the four surgical parameters the surgeon needs to choose when performing SAC (Fig. 3): anterior–posterior and lateral dimensions of the craniotomy, the position of the anterior spring and distance between the anterior and posterior springs, all normalised for pre-operative head size. Here we focused on the effect of these parameters on the correction of head shape features associated with sagittal synostosis, hence considering to be “most successful” the options that resulted in biggest overall bi-parietal widening with a reduction of the posterior narrowing (top view Fig. 1b).

Small *AP* resulted in prominences on top of the head (lateral view Fig. 4b) and bullet-like head shapes with posterior narrowing (top view Fig. 4b), which suggests that small values of *AP* may restrict the adaptation of parietal bone to spring openings leading to localised changes. This might be due to greater amounts of bone with the fused suture being discarded when *AP* values are big, thus allowing more changes to occur. At the same time, the width of parasagittal osteotomies (*LAT*) determined the initial spring opening. Since the forces that the compressed springs exert on the skull bone are proportional to their opening (from high forces for small openings to zero force once the springs open fully), smaller *LAT *values resulted in bigger forces and thus more effective widening of the head (Fig. 5b).

As far as spring position is concerned, surgeons often place the anterior spring close to the coronal suture (small *CorToAnt*) with the objective of reducing the patient’s prominent forehead (referred to as frontal bossing). However, Fig. 6 suggests that such practice on average led to lack of widening and a less rounding profile in the posterior part of the head, due to the springs acting mainly on the anterior side of the parietal bone. Lastly, placing both springs close to each other (small *AntToPost*) resulted in localised head shape changes, apparent in the lateral view in Fig. 7. This was most likely because the force imparted by both springs was confined to a smaller portion of the skull, while when springs were further apart, the force distribution reached the whole parietal region.

In summary, results indicate that SAC was most successful (i.e. maximum overall bi-parietal widening was achieved) when the anterior–posterior craniotomy length was big, the width of parasagittal osteotomies was narrow, the anterior spring was positioned far from the coronal suture and the separation between both springs was big. Overall, population-based 3D statistical shape modelling allowed for quantification and visualisation of trends in achieved head shape outcomes depending on each of the selected surgical parameters.

It must be noted that although trends discovered here can already facilitate surgical decisions, surgeons might face physical restrictions when performing SAC. For example, in order to maximise spring opening from insertion to removal and obtain maximum head widening, small values of *LAT* were found to be more effective in our cohort. However, the minimum width between parasagittal osteotomies is restricted by the fact that extreme care must be taken while performing the cuts not to damage the vein that runs below the fused sagittal suture, called the sagittal sinus [57]. Further, localised skull characteristics (such as locally damaged or fragile sites) may obstruct spring positioning within the reported limits.

Therefore, “small” and “big” values of the surgical parameters described in this study should be understood within the limits of values reported in Table 2. Using the reported findings for validation purposes, other methodologies such as finite element modelling (FEM) [58] could provide additional insight into how varying each surgical parameter past such limits may impact on final shape outcome. In addition, FEM analysis would allow a mechanistic interpretation of the results presented here, determining the strains that occur in the skull and sutures both at surgery and during the distraction process. However, creating FEM models would require computed tomography scans of sagittal patients, not routinely acquired in this cohort at our centre, and skull and suture material properties would need to be defined. In this study, we take advantage of radiation-free scanning and population-based statistical analysis to assess the effect of chosen surgical parameters which have been found to be indeed strongly associated with achieved local 3D head shape features in the SAC procedure.

The main limitation of the current study is the relatively small sample size. Future studies should increase the number of patients in order to create more robust predictive models which could also consider factors such as patient age or severity of the pathology when analysing the effect of surgical choices in more detail. However, we believe that our cohort of twenty patients with the same diagnosis of non-syndromic single suture sagittal synostosis who have been operated by the same surgical team following the same protocols is suited well to investigate associations between surgical decisions and outcomes. Despite the small sample size, correlations between *PLS shape vectors* and corresponding surgical parameters were strong and the computed 3D models showed logical trends, in line with changes observed in individual patients and with expert clinical opinion.

With this in mind, we believe that the proposed image-based computational methodology can be applied to other disciplines and surgical procedures for relating surgical parameters and post-operative results—the ultimate aim being facilitating surgical decision making to improve surgical outcome.

## Conclusion

In this study, 3D handheld scanning in combination with computational statistical shape modelling was employed to relate surgical parameters with long-term global and local 3D head shape features in sagittal patients undergoing spring-assisted cranioplasty. Using partial least squares regression, it was found that craniotomy dimensions and position of springs have a significant effect on local 3D head shape features about 4–5 months after initial surgery. The methodology described here could also be implemented for understanding long-term shape implications of cranio-maxillofacial surgery, which are of paramount importance when performing surgery in growing children. In conclusion, this study demonstrated that an image-based computational methodology involving statistical shape modelling and partial least squares regression provides a powerful platform to untangle the average effect of individual surgical choices in order to guide surgeons in optimising their procedural approach.
